# Correlation between dietary inflammation and mortality among hyperlipidemics

**DOI:** 10.1186/s12944-023-01975-0

**Published:** 2023-11-28

**Authors:** Lili Wang, Tao Liu, Qingdui Zhang, Lele Wang, Qiang Zhou, Jing Wang, Hao Miao, Ji Hao, Chunmei Qi

**Affiliations:** 1 Department of Cardiology, The Second Affiliated Hospital of Xuzhou Medical University, 221000 Xuzhou, China; 2 Department of Cardiology, Jinshan Branch of Shanghai Sixth People’s Hospital, 201500 Shanghai, China

**Keywords:** Hyperlipidaemia, Dietary inflammatory index, Risk of death, Cohort study, Adult population

## Abstract

**Background and objective:**

Although the the Dietary Inflammatory Index (DII) serves to be one of the reliable indicator for hyperlipidaemia, there is still uncertainty about its relationship to prognosis in the hyperlipidaemic population. In current study, the DII levels were analyzed in relation to the mortality risk among among the hyperlipidaemic individuals with the aim of determining any prospective correlation.

**Methods:**

14,460 subjects with hyperlipidaemia from the 10-year (2001–2010) National Health and Nutrition Examination Survey (NHANES) were chosen for this study. The endpoint event for follow-up was all-cause mortality, and subjects were tracked for up to December 31, 2019, or death, whichever occurred first. The tertiles of the DII levels were utilized for categorizing the study population into three groups. Survival curves, Cox proportional hazards regression models, restricted cubic spline (RCS), subgroup and interaction analyses, and sensitivity analyses were employed sequentially for the purpose of evaluating the association of the DII with mortality.

**Results:**

3170 (21.92%) all-cause deaths were recorded during an average 148-month follow-up period. Kaplan-Meier survival curves indicated that the survival rate of participants divided into the low DII group was substantially improved compared to that of those in the higher DII group (log-rank *P* < 0.001). After controlling for confounders, higher levels of DII were observed to be meaningfully linked to an elevated risk of death, no matter whether DII was specified for the continuous (hazard ratio (HR): 1.06; 95% confidence interval (CI): 1.04–1.08) or the categorical variable (HR: 1.22; 95% CI: 1.11–1.33). The DII and mortality displayed a linear association, according to the RCS. Stratified and sensitivity analyses reinforced the proof that these findings were reliable.

**Conclusion:**

Among patients with hyperlipidaemia, the risk of death was positively and linearly linked with DII levels.

**Supplementary Information:**

The online version contains supplementary material available at 10.1186/s12944-023-01975-0.

## Introduction

Hyperlipidaemia is a separate and modifiable risk marker of a series of cardiovascular and cerebrovascular disorders, including coronary atherosclerotic heart disease and stroke, and its global prevalence has continued to rise in recent years [1, 2]. It is a disorder of lipid metabolism that mainly consists of elevated levels of plasma cholesterol, triglycerides, and low-density lipoprotein cholesterol (LDL-C), while high-density lipoprotein cholesterol (HDL-C) levels are reduced [3]. Hyperlipidaemia is prevalent worldwide but easily overlooked, and it seriously increases the health care burden, both in developed and low-income countries [4, 5]. Therefore, it is critical to take strong and effective measures to prevent the occurrence of hyperlipidaemia, delay its progression, and improve its survival rate.

Aside from pharmacologic interventions, the rational diet is a powerful means of assisting in the treatment of patients with dyslipidaemia [6]. Unhealthy dietary patterns contribute to the overproduction of pro-inflammatory cytokines from one’s own body while simultaneously decreasing the production of anti-inflammatory factors, which in turn leads the body’s immune response to be chronically activated and the body’s inflammatory burden increases [7, 8]. Additionally, such diets are commonly responsible for diseases such as rheumatoid arthritis, cardiovascular disease, hyperlipidaemia, tumours, and diabetes [9–12]. In contrast, previous studies have found that the Mediterranean dietary pattern provides significant anti-inflammatory effects that are strongly associated with protection against chronic medical conditions [13, 14]. Established researches have primarily focused on addressing the association between particular nutrients or foods and disease, rather than the possibility of inflammation in the entire diet, which might have limited their results. In an effort to improve this situation, the Shivappa team developed the Dietary Inflammatory Index (DII) algorithm that assesses the exact extent of underlying inflammation in the diet of an individual and is built by scoring 45 food components (macronutrients, micronutrients, and other dietary constituents) that have pro-inflammatory or anti-inflammatory properties [15].

DII scores ranging from low to high indicate inflammatory properties ranging from combating inflammation towards promoting inflammation [15]. It is important to note that even if the nutrient utilized for calculating the DII has a score lower than 30, the DII score remains usable and allows for utilization across cultures and dietary patterns [16]. Hypertensive populations, chronic kidney disease (CKD) populations, obese populations, and others have all been validated as being at increased risk of death with elevated DII scores [17, 18]. In spite of the fact that the DII increases the risk of suffering from hyperlipidaemia, but the relevance of the DII to the prognosis of people with hyperlipidaemia remains indistinct [19]. As a result, a nationally representative United States (US) cohort was studied for discovering whether the DII was associated with mortality. Furthermore, with the aim of providing new ideas for the management of hyperlipidaemic populations and the reduction in the mortality associated with hyperlipidaemic, this study was conducted.

## Methods

### Subjects for study

National Health and Nutrition Examination Survey (NHANES) was designed as a cross-sectional study for determining public health and nutrient-related levels among American citizens. Conducted every two years, the survey includes sociodemographic characteristics, dietary and health-related questions, physical examination and laboratory indicators from representative samples of 15 cities (counties) throughout the US. The National Center for Health Statistics Ethics Review Committee authorized the extraction of data as well as use for this project.

**Fig. 1:**
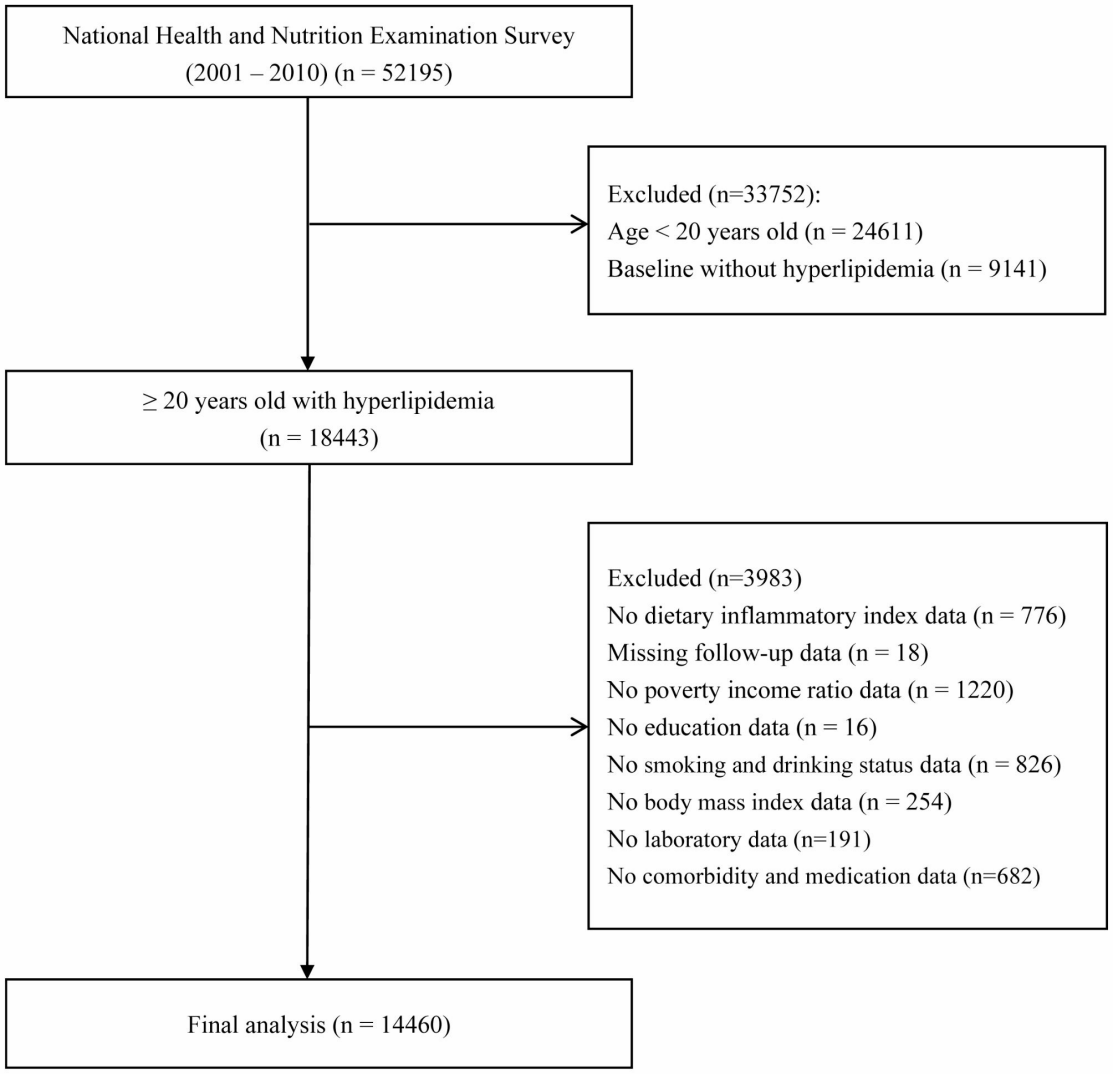
Inclusion and exclusion process of the NHANES 2001–2010

This was a longitudinal analysis that extracted data from the NHANES database for 2001 to 2010 (5 two-year cycles) and integrated them. A total of 51,952 participants who completed medical evaluations at the NHANES ambulatory screening centre were enrolled. The following parameters were applied to determine hyperlipidaemia: LDL-C (≥ 130 mg/dL), total cholesterol (≥ 150 mg/dL), triglycerides (≥ 200 mg/dL), HDL-C (male < 40 mg/dL or female < 50 mg/dL), as well as utilizing blood lipid-lowering medication [20]. After excluding those aged < 20 years (n = 16,078) and those without hyperlipidaemia at baseline (n = 5364), 18,443 hyperlipidemic subjects aged ≥ 20 years remained. Participants with missing DII information (n = 776), missing follow-up data (n = 18), and missing covariate information involved in this study (n = 3189) were further excluded. As shown in Figs. 1, 14 and 460 subjects were ultimately included in this study for analysis.

### Independent variable acquisition

Two 1-day diet recall surveys were employed to collect the dietary data of all participants. In this investigation, the DII score was generated using twenty-eight nutrients for evaluating the degree of potential inflammation in dietary components: total fat, omega-3 fatty acids, niacin, caffeine, energy, fibre, folic acid, partial fat-soluble vitamins (A, D, and E), beta-carotene, partial fat-soluble vitamins (thiamin, pyridoxine, cyanocobalamin, and ascorbic acid), selenium, polyunsaturated fatty acids, Mg, Fe, zinc, omega-6 fatty acids, protein, carbohydrates, alcohol, cholesterol, monounsaturated fatty acids, and saturated fat [15]. The calculation process of the DII was as follows: First, the corresponding nutrients were Z-transformed by comparing the mean and standard deviation of 45 dietary nutrients in the global dietary standard library. After twice the converted value for data centralization, “1” was deducted from the Z-transformed score to get its percentage result. Second, the obtained values were multiplied by the relevant impact scores for achieving the DII score for every kind of nutrient. Third, all food nutrients were totaled for the purpose of determining every individual’s DII score.

### Covariates

The covariates were selected as confounders based on their biological plausibility or prior research investigations. Continuous variables included age (in years) and estimated glomerular filtration rate (eGFR, measuring unit is millilitres per minute per square metre). Categorical variables consisted of sex (composed of male and female), poverty income ratio (PIR, three categories: less than 1.3 for low-income households, between 1.3 and 3.5 for middle-income households, and above 3.5 for high-income households), race (divided into non-Hispanic white, other races, Mexican American, and non-Hispanic black), educational background (three kinds: less than high-school certificate, high-school certificate, and above high-school certificate), smoker (yes or no), drinker (yes or no), body mass index (BMI, three categories: normal weight, overweight, and obesity), hypertension (yes or no), diabetes (yes or no), CKD (yes or no), cardiovascular disease (CVD, yes or no), antihypertensive agent (yes or no), and hypoglycaemic agent (yes or no). Smokers were defined by the interview questions, “Have you smoked 100 or more cigarettes during your entire life?” and “Did you continue to smoke at the time of the interview?”. If both answers were yes, the person was categorized into smokers’ group. Alcohol consumption was determined by the question “Do you drink alcohol at least 12 times a year?” (A 12-ounce beer, four-ounce wine, or one-ounce spirit is regarded as one time) [21]. BMI was computed according to height (meters, m) and weight (kilograms, kg) from the physical examination information (BMI = weight/height^2^, kg/m^2^) and grouped according to criteria appropriate for the US population into normal weight (the value of BMI less than 25), overweight group (the value of BMI from 25 to 29.9), and obese group (the value of BMI equal to or greater than 30). The eGFR was estimated on the basis of creatinine, with reference to the creatinine equation of the Chronic Kidney Disease Epidemiology Collaboration [22]. The definition of hypertension included a self-reported diagnosis of hypertension, a minimum value of 140 mmHg for systolic pressure, and/or a minimum value of 90 mmHg for diastolic pressure, along with administration of medications to control blood pressure. Diabetes was described by possessing at least one of the following characteristics: (1) a response of “yes” to the question regarding having been informed that they were suffering from diabetes, (2) a measured value of fasting plasma glucose equal to or greater than 126 mg/dL, or (3) a measured value of haemoglobin A1c equal to or greater than 6.5% [23]. CVDs consist of heart attacks, coronary cardiac diseases, congestive heart failure, and stroke. Click the link below for more details: https://www.cdc.gov/nchs/nhanes/about_nhanes.htm.

### Outcomes

Linked mortality files for public use were available from the NHANES website, which provided follow-up details since the date of participation in the survey until December 31, 2019. The outcome event in this study was all-cause mortality, that is, death from any cause. Regarding survival duration, if the subjects died before the follow-up deadline, the subjects died before the follow-up deadline, the survival time was the time of death minus the subjects’ baseline inclusion time; if the subjects did not die before the follow-up deadline, survival was the follow-up deadline minus the subjects’ baseline inclusion time.

### Statistical analysis

The data analyses were undertaken according to the NHANES analysis guidelines, following the stratified, complex sample design and calculating sample weights. To obtain overall representative features, continuous parameters are described by weighted mean values (standard errors). Furthermore,

weighted T or weighted ANOVA tests were employed for assessing differences across groups. Categorical factors are represented in weighted frequencies (percentages) and were subjected to the weighted chi-square test. The Kaplan-Meier survival curve was plotted, as well as significance tests were performed by the log-rank test for the purpose of whether there were any differences among the groups of patients. The DII was used to assess its effect on mortality risk by employing multivariate Cox proportional hazards models, and considering the DII as both a continuous and a tertile parameter. All-cause mortality was treated as the dependent factor, meanwhile, the DII served as an independent parameter, and the first tertile group served as the control. Model 1 did not include any corrections; In Model 2, age, sex, and race were corrected; On the basis of Model 2, the third model further corrected for additional confounding factors, such as BMI group, PIR group, smoker, drinker, educational level, eGFR, hypertension, diabetes, CVD, CKD, taking antihypertensive drugs and taking hypoglycaemic drugs. There was a calculation of a weighted hazard ratio (HR) along with its weighted 95% confidence interval (CI). Immediately after that, Model 3 was tested for the presence of multicollinearity by the variance inflation factor (VIF) detection. In addition, a dose-response curve was also constructed using a restricted cubic spline (RCS) function as a way of examining the relationship between DII and the risk of death from all causes among hyperlipidaemic patients, with the same covariates adjusted as in Model 3. To investigate the robustness of the results, age, sex, race, BMI group, PIR group, smoker, drinker, educational level, CVD, diabetes, and hypertension were stratified, as well as explored whether there were interactions between these factors and the effect of the DII on mortality. To minimize potential reverse causality bias, Cox proportional hazards models were conducted again after exclusion of study subjects whose deaths occurred within 3 years of follow-up. For data processing, we selected R Studio (version 4.2.1) and Stata (version 18.0). The result of the test was considered significant as long as the value of P is less than 0.05.

## Results

### Baseline parameters

This study involved totally 14,460 participants, with an average age of 49.22 years, among which 7263 (49.61%) participants were male. The DII values ranged from − 5.28 to 5.42, and the baseline features of the study cohort grouped on the basis of DII tertiles (first tertile: -5.28-0.99; second tertile: 1.00-2.67; third tertile: 2.68–5.42) are detailed in Table 1. In contrast to the first tertile, the third tertile had more female participants, low-income individuals, obese individuals, a higher proportion of black individuals, individuals receiving poor educational attainment, and more current smokers. In addition, the third tertile had a higher likelihood of comorbid conditions such as hypertension, CVD, CKD, and diabetes.

**Table 1:** Weighted baseline characteristics of the study subjects, NHANES 2001–2010

Variable	Dietary Inflammatory Index				*P* value
	Total	1st tertile (-5.28-0.99)	2nd tertile (1.00-2.67)	3rd tertile (2.68–5.42)	
Age, year	49.22 (0.26)	49.35 (0.35)	49.48 (0.30)	48.80 (0.30)	0.120
Sex, n (%)					< 0.001
Female	7197 (50.39)	1832 (38.13)	2380 (50.64)	2985 (64.07)	
Male	7263 (49.61)	2989 (61.87)	2439 (49.36)	1835 (35.93)	
Race, n (%)					< 0.001
White	7771 (74.42)	2715 (76.25)	2575 (74.11)	2481 (72.66)	
Black	2451 (9.05)	623 (6.45)	827 (9.26)	1001 (11.79)	
Mexican	2815 (7.64)	1000 (8.25)	948 (7.81)	867 (6.76)	
Others	1423 (8.89)	483 (9.05)	469 (8.82)	471 (8.78)	
Education level, n (%)					< 0.001
Less than high school	4231 (18.54)	1148 (14.45)	1404 (18.47)	1679 (23.29)	
High school	3582 (26.15)	1096 (22.87)	1150 (24.80)	1336 (31.32)	
Above high school	6647 (55.31)	2577 (62.68)	2265 (56.73)	1805 (45.39)	
BMI group, n (%)					< 0.001
Normal	3405 (25.09)	1208 (26.46)	1094 (24.35)	1103 (24.33)	
Overweight	5354 (36.44)	1888 (38.59)	1832 (37.69)	1634 (32.66)	
Obesity	5701 (38.46)	1725 (34.95)	1893 (37.96)	2083 (43.01)	
PIR group, n (%)					< 0.001
Low income	4117 (19.06)	1095 (14.41)	1306 (17.81)	1716 (25.70)	
Middle income	5640 (36.36)	1747 (31.97)	1912 (36.77)	1981 (40.93)	
High income	4703 (44.57)	1979 (53.62)	1601 (45.42)	1123 (33.37)	
Smoker, n (%)	3177 (23.13)	870 (17.72)	998 (22.36)	1309 (30.13)	< 0.001
Drinker, n (%)	9309 (70.10)	3440 (75.99)	3142 (70.34)	2727 (63.14)	< 0.001
Estimated glomerular filtration rate, ml/min/1.73m^2^	91.37 (0.38)	91.95 (0.43)	90.44 (0.44)	91.70 (0.51)	0.001
Hypertension, n (%)	6881 (41.90)	2156 (40.09)	2335 (42.26)	2390 (43.59)	0.020
Diabetes, n (%)	2791 (14.17)	804 (12.96)	981 (14.81)	1006 (14.86)	0.020
Cardiovascular disease, n (%)	1954 (10.22)	533 (8.36)	666 (10.43)	755 (12.11)	< 0.001
Chronic kidney disease, n (%)	2964 (15.26)	848 (12.67)	1000 (15.51)	1116 (17.93)	< 0.001
Anti-hypertensive drugs, n (%)	5114 (29.80)	1553 (27.47)	1783 (31.34)	1778 (30.79)	< 0.001
Anti-diabetic drugs, n (%)	1660 (8.17)	478 (7.44)	587 (8.75)	595 (8.38)	0.110
All cause mortality, n (%)	3170 (15.86)	878 (12.59)	1107 (16.74)	1185 (18.65)	< 0.001

Continuous variables are described as weighted means (standard errors), and categorical variables are expressed as frequencies (percentages). Abbreviations: BMI, body mass index. PIR, poverty income ratio

Following a mean follow-up of 148 months, an overall number of 3170 (21.92%) all-cause deaths were recorded. Compared to the death subgroup, the survivors were more potentially to have a larger proportion of higher-income individuals, a smaller proportion of white individuals, be younger, have a higher education level, and have a higher proportion of drinkers as well as higher levels of eGFR **(**Table 2), a lower prevalence rate on CVD, CKD, diabetes mellitus, hypertension, and medication use rates. Gender, BMI, and the percentage of smokers were not noticeably different between groups.

**Table 2:** Weighted baseline characteristics according to all-cause mortality, NHANES 2001–2010

Variable	Alive (n = 11,290)	Death (n = 3170)	*P* value
Age, year	45.92 (0.24)	66.73 (0.32)	< 0.001
Sex, n (%)			0.460
Female	5777 (50.52)	1420 (49.67)	
Male	5513 (49.48)	1750 (50.33)	
Race, n (%)			< 0.001
White	5640 (72.89)	2131 (82.54)	
Black	1952 (9.11)	499 (8.76)	
Mexican	2458 (8.46)	357 (3.27)	
Others	1240 (9.54)	183 (5.43)	
Education level, n (%)			< 0.001
Less than high school	3030 (16.38)	1201(30.00)	
High school	2734 (25.49)	848 (29.64)	
Above high school	5526 (58.13)	1121 (40.36)	
BMI group, n (%)			0.140
Normal	2556 (24.80)	849 (26.66)	
Overweight	4127 (36.40)	1227 (36.67)	
Obesity	4607 (38.80)	1094 (36.67)	
PIR group, n (%)			< 0.001
Low income	3099 (18.05)	1018 (24.42)	
Middle income	4198 (34.55)	1442 (45.95)	
High income	3993 (47.39)	710 (29.63)	
Smoker, n (%)	2556 (23.20)	621 (22.78)	0.690
Drinker, n (%)	7774 (73.72)	1535 (50.90)	< 0.001
Estimated glomerular filtration rate, ml/min/1.73m^2^	94.79 (0.38)	73.20 (0.58)	< 0.001
Hypertension, n (%)	4581 (36.71)	2300 (69.48)	< 0.001
Diabetes, n (%)	1724 (11.06)	1067 (30.65)	< 0.001
Cardiovascular disease, n (%)	872 (6.02)	1082 (32.46)	< 0.001
Chronic kidney disease, n (%)	1454 (9.95)	1510 (43.41)	< 0.001
Anti-hypertensive drugs, n (%)	3074 (23.92)	2040 (60.95)	< 0.001
Anti-diabetic drugs, n (%)	992 (6.14)	668 (18.91)	< 0.001

Continuous variables are described as weighted means (standard errors), and categorical variables are expressed as frequencies (percentages). Abbreviations: BMI, body mass index; PIR, poverty income ratio

### Correlation of the DII with mortality based on Kaplan-Meier survival curves

**Fig. 2:**
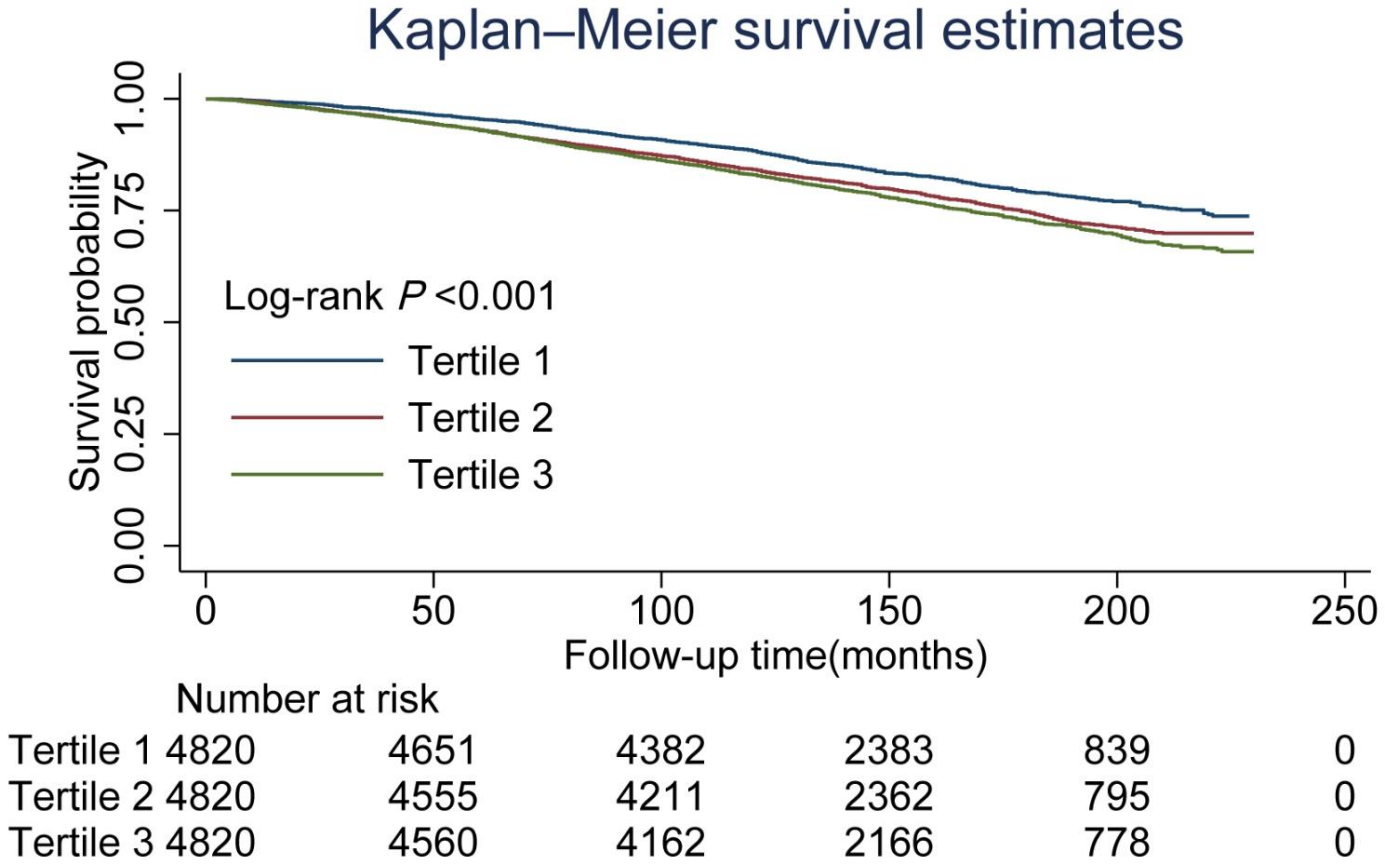
Kaplan-Meier survival curves for all-cause mortality based on the Dietary Inflammatory Index among hyperlipidaemic patients (≥ 20 years old)

As shown by Kaplan-Meier survival curves, the first tertile individuals exhibited a considerably lower risks of dying by any cause compared with second and third tertile individuals (log-rank *P* < 0.05). The DII levels and all-cause mortality revealed powerful hierarchical connections, as displayed in Fig. 2.

### Association of the DII with mortality

The effect on the risk of all-cause death on varying levels of the DII was evaluated utilizing Cox risk-proportional regression models after controlling for relevant confounders as detailed in the Statistical analysis section. As a result of Model 1, compared with the first tertile, both the second and third tertile subgroups showed a significantly increased risk of all-cause death, besides these associations were further strengthened in Model 2 after further adjustment for gender, age, and ethnicity. After fully adjusting for confounding variables, the HRs with corresponding 95% CIs in the second tertile and third tertile groups for all-cause mortality were 1.19 (1.08–1.30) and 1.22 (1.11–1.33), respectively. When the DII level viewed as a continual parameter, Table 3 demonstrates a positive correlation between it and all-cause mortality (HR: 1,06; 95% CI: 1.04–1.08). The VIFs for all variables in Model 3 were all < 5, indicating that there was no multicollinearity among the independent variables (Additional files: Supplementary Tables 1–2).

**Table 3:** Association between the DII levels and all-cause mortality among patients with hyperlipidaemia, NHANES 2001–2010

	Model 1 h (95% CI) *P* value	Model 2 h (95% CI) *P* value	Model 3 h (95% CI) *P* value
Continuous	1.11 (1.08,1.13) < 0.001	1.13 (1.11,1.16) < 0.001	1.06 (1.04,1.08) < 0.001
DII tertile			
1st tertile	ref = 1.00	ref = 1.00	ref = 1.00
2nd tertile	1.34 (1.22,1.47) < 0.001	1.37 (1.26,1.50) < 0.001	1.19 (1.08,1.30) < 0.001
3rd tertile	1.51 (1.38,1.66) < 0.001	1.61 (1.48,1.76) < 0.001	1.22 (1.11,1.33) < 0.001
*P* for trend	< 0.001	< 0.001	< 0.001

Complex sampling weights were considered for all analyses in NHANES.

Abbreviations: DII: Dietary Inflammatory Index; HR, hazard ratio; CI, confidence interval; ref, reference

Model 1: Unadjusted model

Model 2: Adjusted for sex, age and race

Model 3: Confounders such as body mass index, educational level, poverty income ratio, smoker, drinker, estimated glomerular filtration rate, diabetes, hypertension, cardiovascular disease, chronic kidney disease, anti-diabetic drugs, and anti-hypertensive drugs were further adjusted on the basis of Model 2

**Fig. 3:**
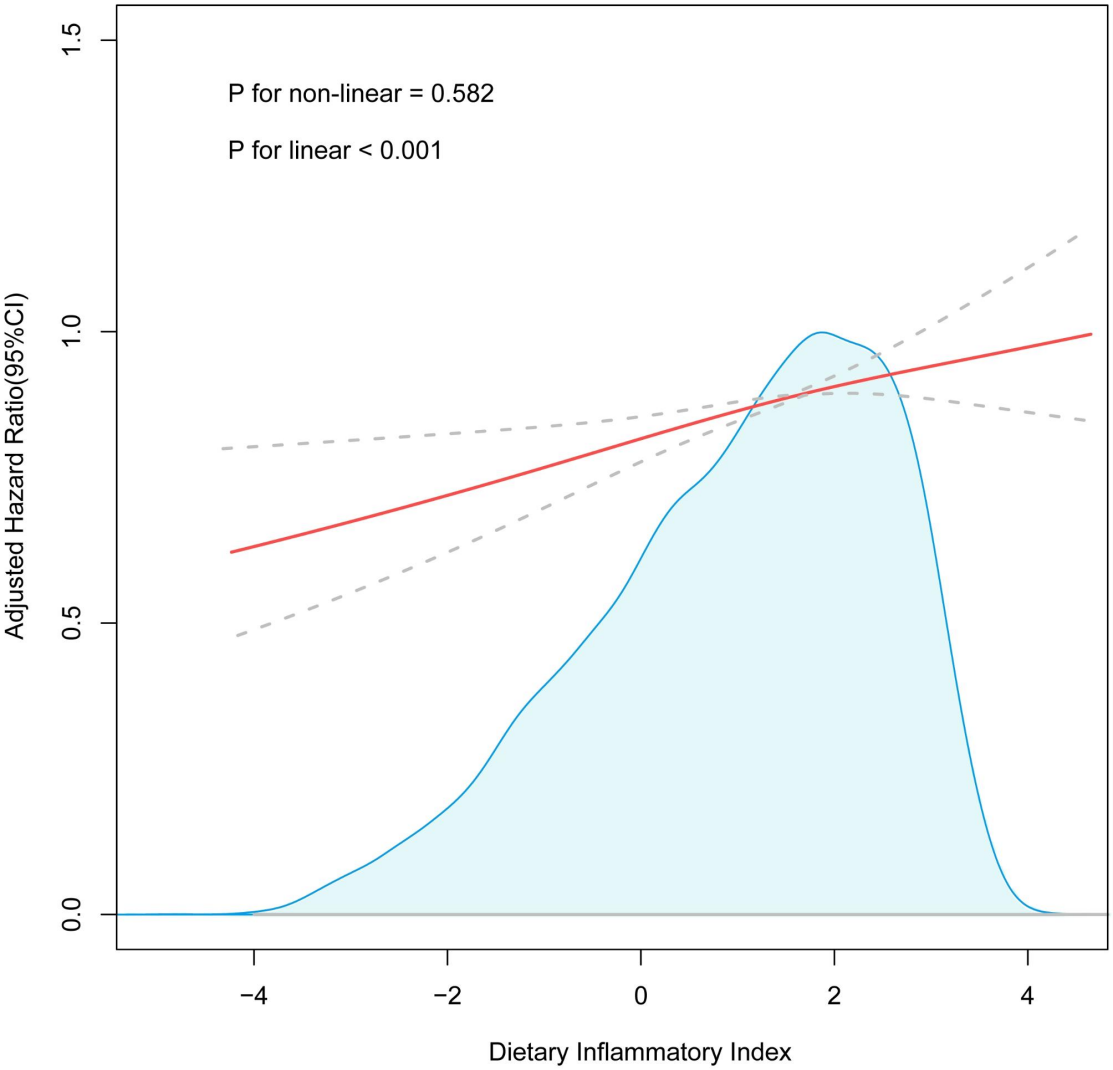
Adjusted cubic spline model of the DII and all-cause mortality among hyperlipidaemic patients (≥ 20 years old) in the NHANES 2001–2010. Adjusted for age, sex, race, BMI, PIR, eGFR, smokers, drinkers, educational level, hypertension, diabetes, CVD, CKD, antihypertensive medications and antidiabetic medications. Abbreviations: DII, Dietary Inflammatory Index; BMI, body mass index; PIR, poverty income ratio; eGFR, estimated glomerular filtration rate; CVD, cardiovascular disease; CKD, chronic kidney disease

The multivariate-corrected RCS results are shown in Fig. 3, suggesting a positive linear correlation between the DII scores of hyperlipidaemic adults and their death risk from all causes (*P* for non-linear = 0.582). Mortality rates across all causes were higher among individuals with elevated DII levels.

### Subgroup analysis and sensitivity analysis

The DII levels and their correlations with the risk of mortality among hyperlipidemic patients were analysed by stratifying for age, gender, race, BMI, PIR, smoker, drinker, educational level, hypertension, diabetes, and CVD (Table 4). There were significant interactions of age, educational level, and diabetes with the associations between the risk by the DII scores and all-cause mortality in hyperlipidaemic patients. The DII level did not interact with any of the other stratification factors. The DII levels and the likelihood of death was essentially unchanged when those who died within three years were excluded, demonstrating that the results were robust (Additional files: Supplementary Figs. 1–2 and Supplementary Table 3).

**Table 4:** Stratified analysis of the DII and risk of death from all causes among patients with hyperlipidaemia, NHANES 2001–2010

	1st tertile HR (95% CI)	2nd tertile HR (95% CI)	3rd tertile HR (95% CI)	*P* for trend	*P* for interaction
Age					0.025
<65	ref = 1.00	1.312 (1.066,1.616)	1.263 (1.020,1.564)	0.044	
≥65	ref = 1.00	1.110 (0.976,1.263)	1.169 (1.032,1.324)	0.015	
Sex					0.876
Female	ref = 1.00	1.231 (1.072,1.413)	1.250 (1.043,1.497)	0.031	
Male	ref = 1.00	1.159 (1.017,1.320)	1.195 (1.043,1.369)	0.007	
Race					0.631
White	ref = 1.00	1.205 (1.082,1.342)	1.245 (1.114,1.393)	< 0.001	
Black	ref = 1.00	1.163 (0.878,1.540)	1.294 (0.993,1.688)	0.048	
Mexican	ref = 1.00	1.086 (0.813,1.451)	0.855 (0.636,1.149)	0.291	
Others	ref = 1.00	1.125 (0.712,1.776)	0.981 (0.541,1.778)	0.934	
Education level					0.036
Less high school	ref = 1.00	0.998 (0.844,1.180)	1.074 (0.929,1.242)	0.291	
High school	ref = 1.00	1.183 (0.968,1.445)	1.186 (1.006,1.398)	0.049	
Above high school	ref = 1.00	1.299 (1.127,1.497)	1.394 (1.177,1.650)	< 0.001	
BMI group					0.816
Normal	ref = 1.00	1.222 (0.984,1.518)	1.303 (1.079,1.573)	0.006	
Overweight	ref = 1.00	1.234 (1.036,1.471)	1.276 (1.093,1.489)	0.002	
Obesity	ref = 1.00	1.134 (0.959,1.340)	1.123 (0.929,1.358)	0.260	
PIR group					0.203
<1.3	ref = 1.00	1.062 (0.884,1.277)	1.193 (0.963,1.478)	0.088	
1.3–3.5	ref = 1.00	1.118 (0.970,1.289)	1.236 (1.077,1.419)	0.003	
>3.5	ref = 1.00	1.368 (1.145,1.633)	1.158 (0.964,1.390)	0.044	
Smoker					0.163
No	ref = 1.00	1.125 (0.858,1.476)	1.005 (0.784,1.290)	0.875	
Yes	ref = 1.00	1.185 (1.070,1.312)	1.288 (1.163,1.427)	< 0.001	
Drinker					0.162
No	ref = 1.00	1.227 (1.059,1.421)	1.163 (0.976,1.387)	0.142	
Yes	ref = 1.00	1.151 (1.010,1.313)	1.299 (1.125,1.499)	< 0.001	
Hypertension					0.387
No	ref = 1.00	1.291 (1.081,1.541)	1.316 (1.093,1.583)	0.003	
Yes	ref = 1.00	1.143 (1.021,1.280)	1.167 (1.034,1.317)	0.016	
Diabetes					0.003
No	ref = 1.00	1.236 (1.101,1.387)	1.339 (1.210,1.482)	< 0.001	
Yes	ref = 1.00	1.068 (0.913,1.249)	0.967 (0.811,1.154)	0.665	
CVD					0.683
No	ref = 1.00	1.213 (1.100,1.337)	1.195 (1.075,1.328)	0.001	
Yes	ref = 1.00	1.132 (0.924,1.386)	1.230 (1.042,1.451)	0.017	

Complex sampling weights were considered for all analyses in NHANES.

Abbreviations: DII: Dietary Inflammatory Index;HR, hazard ratio; CI, confidence interval; ref, reference

Adjusted for sex, age and race, body mass index, educational level, poverty income ratio, estimated glomerular filtration rate, diabetes, hypertension, cardiovascular disease, chronic kidney disease, anti-diabetic drugs, and anti-hypertensive drugs. The calibration factors are excepted from the stratification factor

## Discussion

Using data from a nationally representative cohort, the DII scores and mortality risk among hyperlipidaemics are analyzed for the first time in this study. Treating the DII as a tertile variable revealed that the high DII subgroup exhibited a markedly higher death rate from all causes and a lower survival rate compared with the low DII subgroup. After incorporating the DII and other covariates into a multifactorial Cox proportional hazards model, the risk of death from all causes in the second tertile (HR: 1,22; 95% CI: 1.11–1.33) was 1.22 times higher than that in the first tertile. Consistent findings were observed even though the measure of DII was incorporated to be one continuous parameter: the higher the DII was among adults with hyperlipidaemia in the US, the greater the risk of dying from all causes. The potential positive linear dose‒response association among them was shown through the RCS model.

Prior research has demonstrated that a substantial association exists between higher DII levels and elevated inflammatory factors, especially CRP, IL-6, and so on, which are considered to be the most predictive biomarkers of the inflammatory state in the body [24–26]. Therefore, it could be considered that the DII calculated in this study could well reflect the effect of diet of the study object on the inflammatory state of the body and can be further analysed. In recent years, studies have suggested that higher DII scores are not only strongly linked with an elevated risk of CVD, diabetes, and some common tumours, but also increase the risk of all-cause mortality [27–29]. One study involving 15,291 people with diabetes in the US found that after 45 months of follow-up, diabetic patients on an inflammation-promoting diet (DII > 0) suffered a 71% additional risk of all-cause mortality compared to those (DII < 0) on an inflammation-resisting diet (HR, 1.71; 95% CI, 1.13–2.58; *P* = 0.011) [30]. Furthermore, another report among elderly hypertensive patients in the US observed the same association as described above [17]. A meta-analysis involving 15 studies covering 4 continents illustrated that the RCS intuitively displayed a linear positive dose‒response connection among DII scores and deaths from every cause when the population is no longer confined to one particular country or region [31]. The above-mentioned study findings on the DII in other populations are similar to this study’s results.

Subgroup analyses showed that DII and mortality risk were associated more strongly among adults younger than 65 years (HR: 1.26, 95% CI: 1.02–1.56) than among seniors 65 years and older (HR: 1.17, 95% CI: 1.03–1.32, along with *P* for interaction = 0.025). This may be because older people, who have more comorbid chronic diseases relative to younger people, are more conscious of healthy diets and consume more anti-inflammatory edibles, which results in a weakening impact of the DII on the risk of death [32]. This is often referred to as the reverse causality bias. Similarly, the DII had a more robust influence on death risk among non-diabetics (HR: 1.34, 95% CI: 1.21–1.48 versus HR: 0.97, 95% CI: 0.81–1.15, with *P* for interaction = 0.003) compared with diabetics. This may be a result of the concentration of a high-fibre, and anti-inflammatory dietary pattern in diabetic patients and the anti-inflammatory effects of glucose-lowering medications. This also suggests that non-diabetic people with high DII scores may benefit from dietary interventions much more than those with diabetes.

There is still uncertainty as to why hyperlipidemic patients’ DII scores are associated with death risk. Potential mechanisms include pro-inflammatory diets that increase the levels of inflammatory factors, leading to an imbalance between oxidation and antioxidation in the body, and oxidative stress that accelerates telomere shortening, ultimately exacerbating the onset of ageing and death [33]. In addition, higher DII scores may represent inflammatory factors to activate a series of signalling pathways that drive the development of insulin resistance, which is strongly linked to the occurrence of death [34]. In contrast, the SU.VI.MAX randomized controlled trial reported that supplementation with antioxidants counteracted some of the pro-inflammatory effects of diet, thereby modifying the relationship between DII and mortality, which from another point of view confirms that DII affects body health mainly through inflammation [35].

### Study strengths and limitations

This study showed the following advantages. The information covered in this analysis was obtained from NHANES, which has a large sample size, and the findings are generalizable the whole population of the US, with certain representativeness. Furthermore, this study corrected for numerous potential confounding variables while constructing the sensitivity analysis model for the purpose of confirming the robustness of the findings and reducing the possibility of causal inversion. However, here were still some shortcomings in this study: first, the DII was measured according to a 1-day dietary recall, and recall bias was unavoidable; second, this was an observational study, and causal inferences could not be made; third, while most relevant confounders have been corrected, residual confounders may have remained (e.g., physical activity); and fourth, the present study assessed the initial DII score and the correlation with prognosis, whereas dynamic monitoring of DII scores during follow-up is essential.

## Conclusion

Hyperlipidaemia patients with high DII scores have a greater risk of dying, and the DII score is an independent risk maker of evaluating the prognosis of patients with hyperlipidaemia. This finding also provides data and theoretical support for optimizing dietary structure and establishing an anti-inflammatory dietary concept in hyperlipidaemic populations to reduce the risk of death.

### Electronic supplementary material

Below is the link to the electronic supplementary material.

Supplementary Material 1

Supplementary Material 2

Supplementary Material 3

Supplementary Material 4

Supplementary Material 5

Supplementary Material 6

Supplementary Material 7

## Data Availability

The datasets supporting the conclusions of this article are available on the NHANES website: https://wwwn.cdc.gov/Nchs/Nhanes/.

## Abbreviations

BMI: body mass index
CI: confidence interval
CKD: chronic kidney disease
CVD: cardiovascular disease
DII: Dietary Inflammatory Index
eGFR: estimated glomerular filtration rate
HR: hazard ratio
HDL-C: high-density lipoprotein cholesterol
kg: kilograms
LDL-C: low-density lipoprotein cholesterol
m: metre
NHANES: National Health and Nutrition Examination Survey
PIR: poverty income ratio
RCS: restricted cubic spline
Ref: reference
US: United States
VIF: variance inflation factor
