# Supplementary material for: Correlation between dietary inflammation and mortality among hyperlipidemics
Source: Lipids Health Dis. 2023 Nov 28;22:206. doi: 10.1186/s12944-023-01975-0 (PMC10683303; doi:10.1186/s12944-023-01975-0)
Supplement: Supplementary file 2 — Supplementary Material 2 [file 12944_2023_1975_MOESM2_ESM.docx]

**Supplementary Table 2** Multicollinearity tests based on Model 3 (DII regarded as a categorical variable)

| **Variable** | **GVIF** | **Df** | **GVIF^(1/(2*Df))** |
| --- | --- | --- | --- |
| DII | 1.097 | 2 | 1.023 |
| Age | 1.820 | 1 | 1.349 |
| Sex | 1.099 | 1 | 1.048 |
| Race | 1.288 | 3 | 1.043 |
| Education level | 1.339 | 2 | 1.076 |
| BMI group | 1.160 | 2 | 1.038 |
| PIR group | 1.300 | 2 | 1.068 |
| Smoker | 1.216 | 1 | 1.103 |
| Drinker | 1.166 | 1 | 1.080 |
| eGFR | 2.110 | 1 | 1.452 |
| Hypertension | 1.341 | 1 | 1.158 |
| Diabetes | 2.177 | 1 | 1.475 |
| Cardiovascular disease | 1.188 | 1 | 1.090 |
| Chronic kidney disease | 1.582 | 1 | 1.258 |
| Anti-hypertensive drug | 1.544 | 1 | 1.242 |
| Anti-diabetic drug | 2.160 | 1 | 1.470 |

Abbreviations: BMI, body mass index. DII, Dietary Inflammatory Index; PIR, poverty income ratio; eGFR, estimated glomerular filtration rate.
