# Supplementary material for: Correlation between dietary inflammation and mortality among hyperlipidemics
Source: Lipids Health Dis. 2023 Nov 28;22:206. doi: 10.1186/s12944-023-01975-0 (PMC10683303; doi:10.1186/s12944-023-01975-0)
Supplement: Supplementary file 3 — Supplementary Material 3 [file 12944_2023_1975_MOESM3_ESM.docx]

**Supplementary Table 3** The DII levels and their correlations with the risk of death after eliminating individuals who died during the three-year follow-up period (n = 14088)

|  | **Model 1**  **HR (95% CI) *P* value** | **Model 2**  **HR (95% CI) *P* value** | **Model 3**  **HR (95% CI) *P* value** |
| --- | --- | --- | --- |
| Continuous | 1.10 (1.07,1.12) < 0.001 | 1.13(1.10,1.15) < 0.001 | 1.05(1.03,1.08) < 0.001 |
| DII tertile |  |  |  |
| 1st tertile | ref = 1.00 | ref = 1.00 | ref = 1.00 |
| 2nd tertile | 1.31 (1.18,1.46) < 0.001 | 1.34(1.20,1.49) < 0.001 | 1.17(1.05,1.31) < 0.001 |
| 3rd tertile | 1.47 (1.32,1.63) < 0.001 | 1.58(1.44,1.73) < 0.001 | 1.21(1.10,1.33) < 0.001 |
| *P* for trend | < 0.001 | < 0.001 | < 0.001 |

Complex sampling weights were considered for all analyses in NHANES.

HR, hazard ratio; CI, confidence interval; ref, reference.

Model 1: Unadjusted model.

Model 2: Adjusted for sex, age and race.

Model 3: Confounders such as body mass index, educational level, poverty income ratio, smoker, drinker, estimated glomerular filtration rate, diabetes, hypertension, cardiovascular disease, chronic kidney disease, anti-diabetic drugs, and anti-hypertensive drugs were further adjusted on the basis of Model 2.
