# Supplementary figures and images for: Correlation between dietary inflammation and mortality among hyperlipidemics
Source: Lipids Health Dis. 2023 Nov 28;22:206. doi: 10.1186/s12944-023-01975-0 (PMC10683303; doi:10.1186/s12944-023-01975-0)

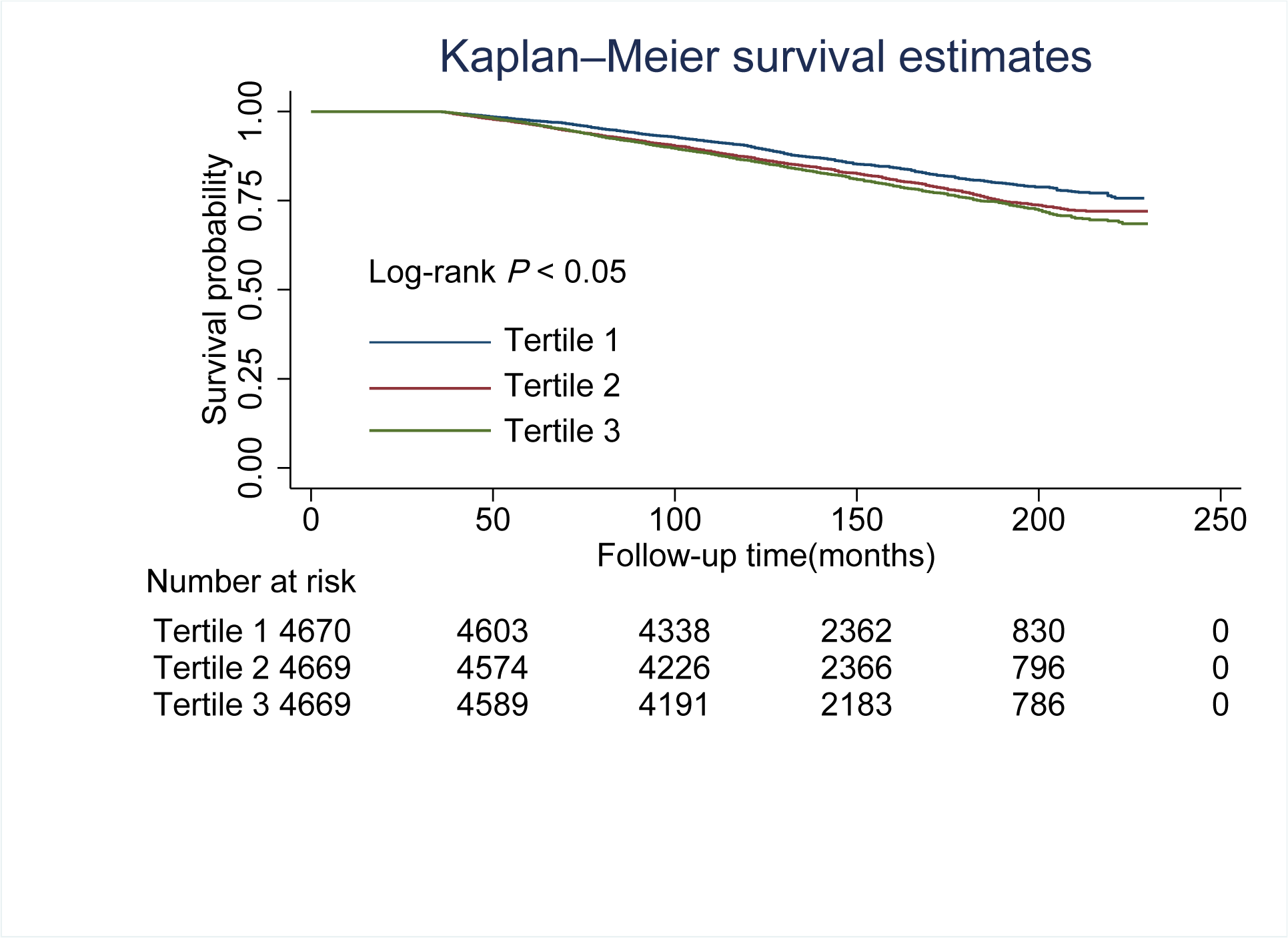

Supplement: Supplementary file 4 — Supplementary Material 4 [file 12944_2023_1975_MOESM4_ESM.tif]

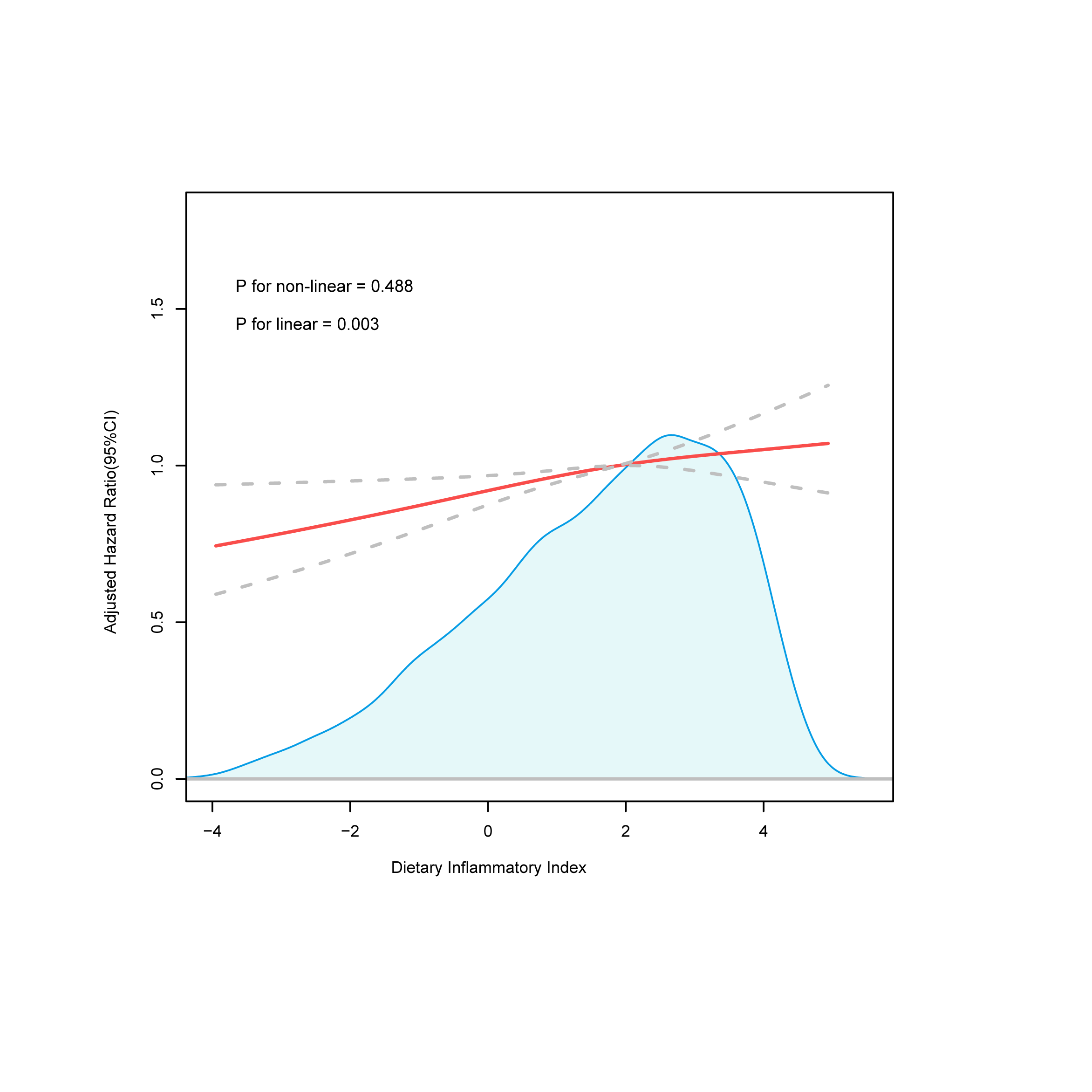

Supplement: Supplementary file 5 — Supplementary Material 5 [file 12944_2023_1975_MOESM5_ESM.tif]
