# Supplementary material for: Correlation between dietary inflammation and mortality among hyperlipidemics
Source: Lipids Health Dis. 2023 Nov 28;22:206. doi: 10.1186/s12944-023-01975-0 (PMC10683303; doi:10.1186/s12944-023-01975-0)
Supplement: Supplementary file 6 — Supplementary Material 6 [file 12944_2023_1975_MOESM6_ESM.pdf]

20231118145050923266072286490624

WORD COUNT 4987

TIME SUBMITTED 18-NOV-2023 02:51PM

PAPER ID 104341467

Correlation between dietary inflammation and mortality among hyperlipidemics

Abstract

Background and objective Although the the Dietary Inflammatory Index (DII) serves to be one of the reliable indicator for hyperlipidaemia, there is still uncertainty about its relationship to prognosis in the hyperlipidaemic population. In current study, the DII levels were analyzed in relation to the mortality risk among among the hyperlipidaemic individuals with the aim of determining any prospective correlation.

Methods 14460 subjects with hyperlipidaemia from the 10-year (2001-2010) National Health and Nutrition Examination Survey (NHANES) were chosen for this study. The endpoint event for follow-up was all-cause mortality, and subjects were tracked for up to December 31, 2019, or death, whichever occurred first. The tertiles of the DII levels were utilized for categorizing the study population into three groups. Survival curves, Cox proportional hazards regression models, restricted cubic spline (RCS), subgroup and interaction analyses, and sensitivity analyses were employed sequentially for the purpose of evaluating the association of the DII with mortality.

Results 3170 (21.92%) all-cause deaths were recorded during an average 148-month follow-up period. Kaplan−Meier survival curves indicated that the survival rate of participants divided into the low DII group was substantially improved compared to that of those in the higher DII group (log-rank $P < 0.001$). After controlling for confounders, higher levels of DII were observed to be meaningfully linked to an elevated risk of death, no matter whether DII was specified for the continuous (hazard ratio (HR): 1.06;

95% confidence interval (CI): 1.04-1.08) or the categorical variable (HR: 1.22; 95% CI: 1.11-1.33). The DII and mortality displayed a linear association, according to the RCS. Stratified and sensitivity analyses reinforced the proof that these findings were reliable.

Conclusion Among patients with hyperlipidaemia, the risk of death was positively and linearly linked with DII levels.

Keywords Hyperlipidaemia, Dietary Inflammatory Index, Risk of death, Cohort study, Adult population

Introduction

Hyperlipidaemia is a separate and modifiable risk marker of a series of cardiovascular and cerebrovascular disorders, including coronary atherosclerotic heart disease and stroke, and its global prevalence has continued to rise in recent years [1, 2]. It is a disorder of lipid metabolism that mainly consists of elevated levels of plasma cholesterol, triglycerides, and low-density lipoprotein cholesterol (LDL-C), while high-density lipoprotein cholesterol (HDL-C) levels are reduced [3]. Hyperlipidaemia is prevalent worldwide but easily overlooked, and it seriously increases the health care burden, both in developed and low-income countries [4, 5]. Therefore, it is critical to take strong and effective measures to prevent the occurrence of hyperlipidaemia, delay its progression, and improve its survival rate.

Aside from pharmacologic interventions, the rational diet is a powerful means of assisting in the treatment of patients with dyslipidaemia [6]. Unhealthy dietary patterns contribute to the overproduction of pro-inflammatory cytokines from one's own body while simultaneously decreasing the production of anti-inflammatory factors, which in turn leads the body's immune response to be chronically activated

and the body's inflammatory burden increases [7, 8]. Additionally, such diets are commonly responsible for diseases such as rheumatoid arthritis, cardiovascular disease, hyperlipidaemia, tumours, and diabetes [9-12]. In contrast, previous studies have found that the Mediterranean dietary pattern provides significant anti-inflammatory effects that are strongly associated with protection against chronic medical conditions [13, 14]. Established researches have primarily focused on addressing the association between particular nutrients or foods and disease, rather than the possibility of inflammation in the entire diet, which might have limited their results. In an effort to improve this situation, the Shivappa team developed the Dietary Inflammatory Index (DII) algorithm that assesses the exact extent of underlying inflammation in the diet of an individual and is built by scoring 45 food components (macronutrients, micronutrients, and other dietary constituents) that have pro-inflammatory or anti-inflammatory properties [15].

DII scores ranging from low to high indicate inflammatory properties ranging from combating inflammation towards promoting inflammation [15]. It is important to note that even if the nutrient utilized for calculating the DII has a score lower than 30, the DII score remains usable and allows for utilization across cultures and dietary patterns [16]. Hypertensive populations, chronic kidney disease (CKD) populations, obese populations, and others have all been validated as being at increased risk of death with elevated DII scores [17, 18]. In spite of the fact that the DII increases the risk of suffering from hyperlipidaemia, but the relevance of the DII to the prognosis of people with hyperlipidaemia remains indistinct [19]. As a result, a nationally representative United States (US) cohort was studied for discovering whether the DII was associated with mortality. Furthermore, with the aim of providing new ideas for the management of hyperlipidaemic populations and the reduction in the mortality associated with hyperlipidaemic, this study was conducted..

Methods

Subjects for Study

National Health and Nutrition Examination Survey (NHANES) was designed as a cross-sectional study for determining public health and nutrient-related levels among American citizens. Conducted every two years, the survey includes sociodemographic characteristics, dietary and health-related questions, physical examination and laboratory indicators from representative samples of 15 cities (counties) throughout the US. The National Center for Health Statistics Ethics Review Committee authorized the extraction of data as well as use for this project.

This was a longitudinal analysis that extracted data from the NHANES database for 2001 to 2010 (5 two-year cycles) and integrated them. A total of 51952 participants who completed medical evaluations at the NHANES ambulatory screening centre were enrolled. The following parameters were applied to determine hyperlipidaemia: LDL-C (≥130 mg/dL), total cholesterol (≥150 mg/dL), triglycerides (≥200 mg/dL), HDL-C (male <40 mg/dL or female <50 mg/dL), as well as utilizing blood lipid-lowering medication [20]. After excluding those aged <20 years (n =16078) and those without hyperlipidaemia at baseline (n = 5364), 18443 hyperlipidemic subjects aged ≥20 years remained. Participants with missing DII information (n =776), missing follow-up data (n =18), and missing covariate information involved in this study (n = 3189) were further excluded. As shown in **Figure 1**, 14,460 subjects were ultimately included in this study for analysis.

Independent variable acquisition

Two 1-day diet recall surveys were employed to collect the dietary data of all participants. In this

investigation, the DII score was generated using twenty-eight nutrients for evaluating the degree of potential inflammation in dietary components: total fat, omega-3 fatty acids, niacin, caffeine, energy, fibre, folic acid, partial fat-soluble vitamins (A, D, and E), beta-carotene, partial fat-soluble vitamins (thiamin, pyridoxine, cyanocobalamin, and ascorbic acid), selenium, polyunsaturated fatty acids, Mg, Fe, zinc, omega-6 fatty acids, protein, carbohydrates, alcohol, cholesterol, monounsaturated fatty acids, and saturated fat [15]. The calculation process of the DII was as follows: First, the corresponding nutrients were Z-transformed by comparing the mean and standard deviation of 45 dietary nutrients in the global dietary standard library. After twice the converted value for data centralization, "1" was deducted from the Z-transformed score to get its percentage result. Second, the obtained values were multiplied by the relevant impact scores for achieving the DII score for every kind of nutrient. Third, all food nutrients were totaled for the purpose of determining every individual's DII score.

Covariates

The covariates were selected as confounders based on their biological plausibility or prior research investigations. Continuous variables included age (in years) and estimated glomerular filtration rate (eGFR, measuring unit is millilitres per minute per square metre). Categorical variables consisted of sex (composed of male and female), poverty income ratio (PIR, three categories: less than 1.3 for low-income households, between 1.3 and 3.5 for middle-income households, and above 3.5 for high-income households), race (divided into non-Hispanic white, other races, Mexican American, and non-Hispanic black), educational background (three kinds: less than high-school certificate, high-school certificate, and above high-school certificate), smoker (yes or no), drinker (yes or no), body mass index (BMI, three categories: normal weight, overweight, and obesity), hypertension (yes or no), diabetes (yes or no), CKD

(yes or no), cardiovascular disease (CVD, yes or no), antihypertensive agent (yes or no), and hypoglycaemic agent (yes or no). Smokers were defined by the interview questions, “Have you smoked 100 or more cigarettes during your entire life?” and “Did you continue to smoke at the time of the interview?”. If both answers were yes, the person was categorized into smokers' group. Alcohol consumption was determined by the question “Do you drink alcohol at least 12 times a year?” (A 12-ounce beer, four-ounce wine, or one-ounce spirit is regarded as one time) [21]. BMI was computed according to height (meters, m) and weight (kilograms, kg) from the physical examination information (BMI = weight/height^2, kg/m^2) and grouped according to criteria appropriate for the US population into normal weight (the value of BMI less than 25), overweight group (the value of BMI from 25 to 29.9), and obese group (the value of BMI equal to or greater than 30). The eGFR was estimated on the basis of creatinine, with reference to the creatinine equation of the Chronic Kidney Disease Epidemiology Collaboration [22]. The definition of hypertension included a self-reported diagnosis of hypertension, a minimum value of 140 mmHg for systolic pressure, and/or a minimum value of 90 mmHg for diastolic pressure, along with administration of medications to control blood pressure. Diabetes was described by possessing at least one of the following characteristics: 1) a response of "yes" to the question regarding having been informed that they were suffering from diabetes, 2) a measured value of fasting plasma glucose equal to or greater than 126 mg/dL, or 3) a measured value of haemoglobin A1c equal to or greater than 6.5% [23]. CVDs consist of heart attacks, coronary cardiac diseases, congestive heart failure, and stroke. Click the link below for more details: https://www.cdc.gov/nchs/nhanes/about_nhanes.htm.

Outcomes

Linked mortality files for public use were available from the NHANES website, which provided follow-

up details since the date of participation in the survey until December 31, 2019. The outcome event in this study was all-cause mortality, that is, death from any cause. Regarding survival duration, if the subjects died before the follow-up deadline, the subjects died before the follow-up deadline, the survival time was the time of death minus the subjects' baseline inclusion time; if the subjects did not die before the follow-up deadline, survival was the follow-up deadline minus the subjects' baseline inclusion time.

Statistical analysis

The data analyses were undertaken according to the NHANES analysis guidelines, following the stratified, complex sample design and calculating sample weights. To obtain overall representative features, continuous parameters are described by weighted mean values (standard errors). Furthermore, weighted T or weighted ANOVA tests were employed for assessing differences across groups. Categorical factors are represented in weighted frequencies (percentages) and were subjected to the weighted chi-square test. The Kaplan-Meier survival curve was plotted, as well as significance tests were performed by the log-rank test for the purpose of whether there were any differences among the groups of patients. The DII was used to assess its effect on mortality risk by employing multivariate Cox proportional hazards models, and considering the DII as both a continuous and a tertile parameter. All-cause mortality was treated as the dependent factor, meanwhile, the DII served as an independent parameter, and the first tertile group served as the control. Model 1 did not include any corrections; In Model 2, age, sex, and race were corrected; On the basis of Model 2, the third model further corrected for additional confounding factors, such as BMI group, PIR group, smoker, drinker, educational level, eGFR, hypertension, diabetes, CVD, CKD, taking antihypertensive drugs and taking hypoglycaemic drugs. There was a calculation of a weighted hazard ratio (HR) along with its weighted 95% confidence

interval (CI). Immediately after that, Model 3 was tested for the presence of multicollinearity by the variance inflation factor (VIF) detection. In addition, a dose-response curve was also constructed using a restricted cubic spline (RCS) function as a way of examining the relationship between DII and the risk of death from all causes among hyperlipidaemic patients, with the same covariates adjusted as in Model 3. To investigate the robustness of the results, age, sex, race, BMI group, PIR group, smoker, drinker, educational level, CVD, diabetes, and hypertension were stratified, as well as explored whether there were interactions between these factors and the effect of the DII on mortality. To minimize potential reverse causality bias, Cox proportional hazards models were conducted again after exclusion of study subjects whose deaths occurred within 3 years of follow-up. For data processing, we selected R Studio (version 4.2.1) and Stata (version 18.0). The result of the test was considered significant as long as the value of P is less than 0.05.

Results

Baseline Parameters

This study involved totally 14460 participants, with an average age of 49.22 years, among which 7263 (49.61%) participants were male. The DII values ranged from -5.28 to 5.42, and the baseline features of the study cohort grouped on the basis of DII tertiles (first tertile: -5.28-0.99; second tertile: 1.00-2.67; third tertile: 2.68-5.42) are detailed in **Table 1**. In contrast to the first tertile, the third tertile had more female participants, low-income individuals, obese individuals, a higher proportion of black individuals, individuals receiving poor educational attainment, and more current smokers. In addition, the third tertile had a higher likelihood of comorbid conditions such as hypertension, CVD, CKD, and diabetes.

Following a mean follow-up of 148 months, an overall number of 3170 (21.92%) all-cause deaths were recorded. Compared to the death subgroup, the survivors were more potentially to have a larger proportion of higher-income individuals, a smaller proportion of white individuals, be younger, have a higher education level, and have a higher proportion of drinkers as well as higher levels of eGFR **(Table 2**), a lower prevalence rate on CVD, CKD, diabetes mellitus, hypertension, and medication use rates. Gender, BMI, and the percentage of smokers were not noticeably different between groups.

Correlation of the DII with mortality based on Kaplan–Meier survival curves

As shown by Kaplan−Meier survival curves, the first tertile individuals exhibited a considerably lower risks of dying by any cause compared with second and third tertile individuals (log-rank $P < 0.05$). The DII levels and all-cause mortality revealed powerful hierarchical connections, as displayed in **Figure 2**.

Association of the DII with mortality

The effect on the risk of all-cause death on varying levels of the DII was evaluated utilizing Cox risk-proportional regression models after controlling for relevant confounders as detailed in the Statistical analysis section. As a result of Model 1, compared with the first tertile, both the second and third tertile subgroups showed a significantly increased risk of all-cause death, besides these associations were further strengthened in Model 2 after further adjustment for gender, age, and ethnicity. After fully adjusting for confounding variables, the HRs with corresponding 95% CIs in the second tertile and third tertile groups for all-cause mortality were 1.19 (1.08-1.30) and 1.22 (1.11-1.33), respectively. When the DII level viewed as a continual parameter, **Table 3** demonstrates a positive correlation between it and

all-cause mortality (HR: 1,06; 95% CI: 1.04-1.08). The VIFs for all variables in Model 3 were all <5, indicating that there was no multicollinearity among the independent variables (**Additional files: Supplementary Table 1- 2**).

The multivariate-corrected RCS results are shown in **Figure 3,** suggesting a positive linear correlation between the DII scores of hyperlipidaemic adults and their death risk from all causes (*P* for non-linear = 0.582). Mortality rates across all causes were higher among individuals with elevated DII levels.

Subgroup analysis and sensitivity analysis

The DII levels and their correlations with the risk of mortality among hyperlipidemic patients were analysed by stratifying for age, gender, race, BMI, PIR, smoker, drinker, educational level, hypertension, diabetes, and CVD (**Table 4**). There were significant interactions of age, educational level, and diabetes with the associations between the risk by the DII scores and all-cause mortality in hyperlipidaemic patients. The DII level did not interact with any of the other stratification factors. The DII levels and the likelihood of death was essentially unchanged when those who died within three years were excluded, demonstrating that the results were robust (**Additional files: Supplementary Figure 1-2 and Supplementary Table 3**).

Discussion

Using data from a nationally representative cohort, the DII scores and mortality risk among hyperlipidaemics are analyzed for the first time in this study. Treating the DII as a tertile variable revealed

that the high DII subgroup exhibited a markedly higher death rate from all causes and a lower survival rate compared with the low DII subgroup. After incorporating the DII and other covariates into a multifactorial Cox proportional hazards model, the risk of death from all causes in the second tertile (HR: 1,22; 95% CI: 1.11-1.33) was 1.22 times higher than that in the first tertile. Consistent findings were observed even though the measure of DII was incorporated to be one continuous parameter: the higher the DII was among adults with hyperlipidaemia in the US, the greater the risk of dying from all causes. The potential positive linear dose–response association among them was shown through the RCS model.

Prior research has demonstrated that a substantial association exists between higher DII levels and elevated inflammatory factors, especially CRP, IL-6, and so on, which are considered to be the most predictive biomarkers of the inflammatory state in the body[24-26]. Therefore, it could be considered that the DII calculated in this study could well reflect the effect of diet of the study object on the inflammatory state of the body and can be further analysed. In recent years, studies have suggested that higher DII scores are not only strongly linked with an elevated risk of CVD, diabetes, and some common tumours, but also increase the risk of all-cause mortality[27-29]. One study involving 15291 people with diabetes in the US found that after 45 months of follow-up, diabetic patients on an inflammation-promoting diet (DII >0) suffered a 71% additional risk of all-cause mortality compared to those (DII <0) on an inflammation-resisting diet (HR, 1.71; 95% CI, 1.13-2.58; $P = 0.011$)[30]. Furthermore, another report among elderly hypertensive patients in the US observed the same association as described above[17]. A meta-analysis involving 15 studies covering 4 continents illustrated that the RCS intuitively displayed a linear positive dose–response connection among DII scores and deaths from every cause when the population is no longer confined to one particular country or region [31]. The above-mentioned

study findings on the DII in other populations are similar to this study's results.

Subgroup analyses showed that DII and mortality risk were associated more strongly among adults younger than 65 years (HR: 1.26, 95% CI: 1.02-1.56) than among seniors 65 years and older (HR: 1.17, 95% CI: 1.03-1.32, along with *P* for interaction = 0.025). This may be because older people, who have more comorbid chronic diseases relative to younger people, are more conscious of healthy diets and consume more anti-inflammatory edibles, which results in a weakening impact of the DII on the risk of death[32]. This is often referred to as the reverse causality bias. Similarly, the DII had a more robust influence on death risk among non-diabetics (HR: 1.34, 95% CI: 1.21-1.48 versus HR: 0.97, 95% CI: 0.81-1.15, with *P* for interaction = 0.003) compared with diabetics. This may be a result of the concentration of a high-fibre, and anti-inflammatory dietary pattern in diabetic patients and the anti-inflammatory effects of glucose-lowering medications. This also suggests that non-diabetic people with high DII scores may benefit from dietary interventions much more than those with diabetes.

There is still uncertainty as to why hyperlipidemic patients' DII scores are associated with death risk. Potential mechanisms include pro-inflammatory diets that increase the levels of inflammatory factors, leading to an imbalance between oxidation and antioxidation in the body, and oxidative stress that accelerates telomere shortening, ultimately exacerbating the onset of ageing and death[33]. In addition, higher DII scores may represent inflammatory factors to activate a series of signalling pathways that drive the development of insulin resistance, which is strongly linked to the occurrence of death[34]. In contrast, the SU.VI.MAX randomized controlled trial reported that supplementation with antioxidants counteracted some of the pro-inflammatory effects of diet, thereby modifying the relationship between

DII and mortality, which from another point of view confirms that DII affects body health mainly through inflammation[35].

Study strengths and limitations

This study showed the following advantages. The information covered in this analysis was obtained from NHANES, which has a large sample size, and the findings are generalizable the whole population of the US, with certain representativeness. Furthermore, this study corrected for numerous potential confounding variables while constructing the sensitivity analysis model for the purpose of confirming the robustness of the findings and reducing the possibility of causal inversion. However, here were still some shortcomings in this study: first, the DII was measured according to a 1-day dietary recall, and recall bias was unavoidable; second, this was an observational study, and causal inferences could not be made; third, while most relevant confounders have been corrected, residual confounders may have remained (e.g., physical activity); and fourth, the present study assessed the initial DII score and the correlation with prognosis, whereas dynamic monitoring of DII scores during follow-up is essential.

Conclusion

Hyperlipidaemia patients with high DII scores have a greater risk of dying, and the DII score is an independent risk maker of evaluating the prognosis of patients with hyperlipidaemia. This finding also provides data and theoretical support for optimizing dietary structure and establishing an anti-inflammatory dietary concept in hyperlipidaemic populations to reduce the risk of death.

References

1. Alloubani A, Nimer R, Samara R: **Relationship between Hyperlipidemia, Cardiovascular Disease and Stroke: A Systematic Review**. *Curr Cardiol Rev* 2021, **17:**e051121189015.
2. Lu Y, Li SX, Liu Y, Rodriguez F, Watson KE, Dreyer RP, Khera R, Murugiah K, D'Onofrio G, Spatz ES, et al: **Sex-Specific Risk Factors Associated With First Acute Myocardial Infarction in Young Adults**. *JAMA Netw Open* 2022, **5:**e229953.
3. Hill MF, Bordoni B. Hyperlipidemia. 2023. Treasure Island (FL).
4. Karr S: **Epidemiology and management of hyperlipidemia**. *Am J Manag Care* 2017, **23:**S139-S148.
5. Du H, Shi Q, Song P, Pan XF, Yang X, Chen L, He Y, Zong G, Zhu Y, Su B, Li S: **Global Burden Attributable to High Low-Density Lipoprotein-Cholesterol From 1990 to 2019**. *Front Cardiovasc Med* 2022, **9:**903126.
6. Grundy SM, Stone NJ, Bailey AL, Beam C, Birtcher KK, Blumenthal RS, Braun LT, de Ferranti S, Faiella-Tommasino J, Forman DE, et al: **2018 AHA/ACC/AACVPR/AAPA/ABC/ACPM/ADA/AGS/APhA/ASPC/NLA/PCNA Guideline on the Management of Blood Cholesterol: Executive Summary: A Report of the American College of Cardiology/American Heart Association Task Force on Clinical Practice Guidelines**. *Circulation* 2019, **139:**e1046-e1081.
7. Di Giosia P, Stamerra CA, Giorgini P, Jamialahamdi T, Butler AE, Sahebkar A: **The role of nutrition in inflammaging**. *Ageing Res. Rev.* 2022, **77:**101596.
8. Grosso G, Laudisio D, Frias-Toral E, Barrea L, Muscogiuri G, Savastano S, Colao A: **Anti-Inflammatory Nutrients and Obesity-Associated Metabolic-Inflammation: State of the Art and Future Direction**. *Nutrients* 2022, **14**.
9. Wagenaar CA, van de Put M, Bisschops M, Walrabenstein W, de Jonge CS, Herrema H, van Schaardenburg

D: **The Effect of Dietary Interventions on Chronic Inflammatory Diseases in Relation to the Microbiome: A Systematic Review**. *Nutrients* 2021, **13**.

10. Yurtdaş G, Akbulut G, Baran M, Yılmaz C: **The effects of Mediterranean diet on hepatic steatosis, oxidative stress, and inflammation in adolescents with non-alcoholic fatty liver disease: A randomized controlled trial**. *Pediatr Obes* 2022, **17:**e12872.

11. Casas R, Castro-Barquero S, Estruch R, Sacanella E: **Nutrition and Cardiovascular Health**. *Int J Mol Sci* 2018, **19**.

12. Xiang S, Wang Y, Qian S, Li J, Jin Y, Ding X, Xu T: **The association between dietary inflammation index and the risk of rheumatoid arthritis in Americans**. *Clin. Rheumatol.* 2022, **41:**2647-2658.

13. Filippou CD, Thomopoulos CG, Kouremeti MM, Sotiropoulou LI, Nihoyannopoulos PI, Tousoulis DM, Tsioufis CP: **Mediterranean diet and blood pressure reduction in adults with and without hypertension: A systematic review and meta-analysis of randomized controlled trials**. *Clinical nutrition (Edinburgh, Scotland)* 2021, **40:**3191-3200.

14. Li J, Guasch-Ferré M, Chung W, Ruiz-Canela M, Toledo E, Corella D, Bhupathiraju SN, Tobias DK, Tabung FK, Hu J, et al: **The Mediterranean diet, plasma metabolome, and cardiovascular disease risk**. *Eur. Heart J.* 2020, **41:**2645-2656.

15. Shivappa N, Steck SE, Hurley TG, Hussey JR, Hébert JR: **Designing and developing a literature-derived, population-based dietary inflammatory index**. *Public Health Nutr* 2014, **17:**1689-1696.

16. Marx W, Veronese N, Kelly JT, Smith L, Hockey M, Collins S, Trakman GL, Hoare E, Teasdale SB, Wade A, et al: **The Dietary Inflammatory Index and Human Health: An Umbrella Review of Meta-Analyses of Observational Studies**. *Advances in nutrition (Bethesda, Md.)* 2021, **12:**1681-1690.

17. Cao Y, Li P, Zhang Y, Qiu M, Li J, Ma S, Yan Y, Li Y, Han Y: **Dietary Inflammatory Index and All-Cause**

Mortality in Older Adults with Hypertension: Results from NHANES. *J Clin Med* 2023, **12**.

18. Yan LJ, Zhang FR, Ma CS, Zheng Y: **Higher dietary inflammatory index is associated with increased all-cause mortality in adults with chronic kidney disease**. *Front Nutr* 2022, **9:**883838.

19. Han Y, Jiang X, Qin Y, Zhao Y, Zhang G, Liu C: **A cross-sectional study exploring the relationship between the dietary inflammatory index and hyperlipidemia based on the National Health and Nutrition Examination Survey (2005-2018)**. *Lipids Health Dis* 2023, **22:**140.

20. **Third Report of the National Cholesterol Education Program (NCEP) Expert Panel on Detection, Evaluation, and Treatment of High Blood Cholesterol in Adults (Adult Treatment Panel III) final report**. *Circulation* 2002, **106:**3143-3421.

21. Hicks CW, Wang D, Matsushita K, Windham BG, Selvin E: **Peripheral Neuropathy and All-Cause and Cardiovascular Mortality in U.S. Adults : A Prospective Cohort Study**. *Ann. Intern. Med.* 2021, **174:**167-174.

22. Levey AS, Stevens LA, Schmid CH, Zhang YL, Castro AF 3rd, Feldman HI, Kusek JW, Eggers P, Van Lente F, Greene T, Coresh J: **A new equation to estimate glomerular filtration rate**. *Ann. Intern. Med.* 2009, **150:**604-612.

23. **2. Classification and Diagnosis of Diabetes: Standards of Medical Care in Diabetes-2020**. *Diabetes Care* 2020, **43:**S14-S31.

24. Shivappa N, Hebert JR, Marcos A, Diaz LE, Gomez S, Nova E, Michels N, Arouca A, González-Gil E, Frederic G, et al: **Association between dietary inflammatory index and inflammatory markers in the HELENA study**. *Mol Nutr Food Res* 2017, **61**.

25. Tabung FK, Smith-Warner SA, Chavarro JE, Wu K, Fuchs CS, Hu FB, Chan AT, Willett WC, Giovannucci EL: **Development and Validation of an EmPIRical Dietary Inflammatory Index**. *J. Nutr.* 2016,

146:1560-1570.

26. Haß U, Herpich C, Kochlik B, Weber D, Grune T, Norman K: **Dietary Inflammatory Index and Cross-Sectional Associations with Inflammation, Muscle Mass and Function in Healthy Old Adults**. *J Nutr Health Aging* 2022, **26:**346-351.

27. Hariharan R, Odjidja EN, Scott D, Shivappa N, Hébert JR, Hodge A, de Courten B: **The dietary inflammatory index, obesity, type 2 diabetes, and cardiovascular risk factors and diseases**. *Obes Rev* 2022, **23:**e13349.

28. Zhang J, Jia J, Lai R, Wang X, Chen X, Tian W, Liu Q, Li J, Ju J, Xu H: **Association between dietary inflammatory index and atherosclerosis cardiovascular disease in U.S. adults**. *Front Nutr* 2022, **9:**1044329.

29. Gardeazabal I, Ruiz-Canela M, Sánchez-Bayona R, Romanos-Nanclares A, Aramendía-Beitia JM, Shivappa N, Hébert JR, Martínez-González MA, Toledo E: **Dietary inflammatory index and incidence of breast cancer in the SUN project**. *Clinical nutrition (Edinburgh, Scotland)* 2019, **38:**2259-2268.

30. Tan J, Liu N, Sun P, Tang Y, Qin W: **A Proinflammatory Diet May Increase Mortality Risk in Patients with Diabetes Mellitus**. *Nutrients* 2022, **14**.

31. Zhang J, Feng Y, Yang X, Li Y, Wu Y, Yuan L, Li T, Hu H, Li X, Huang H, et al: **Dose-Response Association of Dietary Inflammatory Potential with All-Cause and Cause-Specific Mortality**. *Advances in nutrition (Bethesda, Md.)* 2022, **13:**1834-1845.

32. Hiza HA, Casavale KO, Guenther PM, Davis CA: **Diet quality of Americans differs by age, sex, race/ethnicity, income, and education level**. *J Acad Nutr Diet* 2013, **113:**297-306.

33. Xie R, Ning Z, Xiao M, Li L, Liu M, Zhang Y: **Dietary inflammatory potential and biological aging among US adults: a population-based study**. *Aging Clin Exp Res* 2023, **35:**1273-1281.

34. Shu Y, Wu X, Wang J, Ma X, Li H, Xiang Y: **Associations of Dietary Inflammatory Index With Prediabetes and Insulin Resistance**. *Front Endocrinol (Lausanne)* 2022, **13:**820932.

35. Graffouillère L, Deschasaux M, Mariotti F, Neufcourt L, Shivappa N, Hébert JR, Wirth MD, Latino-Martel P, Hercberg S, Galan P, et al: **Prospective association between the Dietary Inflammatory Index and mortality: modulation by antioxidant supplementation in the SU.VI.MAX randomized controlled trial**. *Am. J. Clin. Nutr.* 2016, **103:**878-885.

Tables

Table 1 Weighted baseline characteristics of the study subjects, NHANES 2001-2010

Table 2 Weighted baseline characteristics according to all-cause mortality, NHANES 2001– 2010

Table 3 Association between the DII levels and all-cause mortality among patients with hyperlipidaemia, NHANES 2001– 2010

Table 4 Stratified analysis of the DII and risk of death from all causes among patients with hyperlipidaemia, NHANES 2001-2010

Figures

Fig. 1 Inclusion and exclusion process of the NHANES 2001-2010

Fig. 2 Kaplan‐Meier survival curves for all-cause mortality based on the Dietary Inflammatory Index among hyperlipidaemic patients (≥ 20 years old)

Fig. 3 Adjusted cubic spline model of the DII and all-cause mortality among hyperlipidaemic patients (≥ 20 years old) in the NHANES 2001-2010.

Additional Files

Supplementary Table 1 Multicollinearity tests based on Model 3 (DII regarded as a continuous variable)

Supplementary Table 2 Multicollinearity tests based on Model 3 (DII regarded as a categorical variable)

Supplementary Table 3 The DII levels and their correlations with the risk of death after eliminating individuals who died during the three-year follow-up period (n = 14088)

Supplementary Fig. 1 Kaplan-Meier survival curves of all-cause mortality based on the Dietary Inflammatory Index among hyperlipidaemic patients after excluding individuals who died within the three-year follow-up period (n = 14088).

Supplementary Fig. 2 Adjusted cubic spline model of the relationship between the Dietary Inflammatory Index and all-cause mortality among hyperlipidaemic patients after excluding participants who died during the three-year follow-up period (n = 14088).

20231118145050923266072286490624

ORIGINALITY REPORT

9%
SIMILARITY INDEX

PRIMARY SOURCES

1	www.omicsdi.org	Internet	34 words — 1%
2	www.science.gov	Internet	30 words — 1%
3	www.frontiersin.org	Internet	28 words — 1%
4	boris.unibe.ch	Internet	23 words — 1%
5	worldwidescience.org	Internet	16 words — < 1%
6	www.wjgnet.com	Internet	16 words — < 1%
7	www.omicsonline.org	Internet	15 words — < 1%
8	Shaowen Zeng, Lei Qi, Yaofei Sun, Guishan Zhuang. "Association of chronic kidney disease with dietary inflammatory index in adults aged 50 years and older: dose-response analysis of a nationally representative population-based study", Journal of Renal Nutrition, 2023	Crossref	14 words — < 1%

9 Song-Yi Park, Chloe P. Lozano, Yurii B. Shvetsov, Carol J. Boushey et al. "Change in the inflammatory potential of diet over 10 years and subsequent mortality: the Multiethnic Cohort Study", British Journal of Nutrition, 2022 — 13 words — < 1%

Crossref

10 vdoc.pub — 13 words — < 1%

Internet

11 Xiaoxu Ren, Xiangchun Liu, Hairong Chen, Rong Wang, Yutian Tian, Ying Zhang, Lifeng Yu, Zhiming Jiang. "Association between riboflavin intake and the risk of all-cause mortality of patients with chronic kidney disease: A retrospective cohort study", Research Square Platform LLC, 2023 — 12 words — < 1%

Crossref Posted Content

12 bmcgeriatr.biomedcentral.com — 11 words — < 1%

Internet

13 lai qiancheng, Liu Ye, Jun Luo, Cheng Zhang, shao yue. "The cross-sectional correlation between the oxidative balance score and cardiometabolic risk factors and its potential correlation with longitudinal mortality in patients with cardiometabolic risk factors.", Cold Spring Harbor Laboratory, 2023 — 11 words — < 1%

Crossref Posted Content

14 Ching-Chi Chi, Yen-Wen Wu, Ting-Hsing Chao, Chih-Chiang Chen et al. "2022 Taiwanese Dermatological Association (TDA), Taiwanese Association for Psoriasis and Skin Immunology (TAPSI), and Taiwan Society of cardiology (TSOC) joint consensus recommendations for the management of psoriatic disease with attention to — 10 words — < 1%

cardiovascular comorbidities", Journal of the Formosan Medical Association, 2022
Crossref

15 breast-cancer-research.biomedcentral.com
Internet
10 words — < 1%

16 www.sgim.org
Internet
10 words — < 1%

17 mdpi.com
Internet
9 words — < 1%

18 translational-medicine.biomedcentral.com
Internet
9 words — < 1%

19 www.amhsr.org
Internet
9 words — < 1%

20 "Biomarkers in Nutrition", Springer Science and Business Media LLC, 2022
Crossref
8 words — < 1%

21 Barbara Dołęgowska, Wojciech Błogowski, Karolina Kędzierska, Krzysztof Safranow et al. "Platelets arachidonic acid metabolism in patients with essential hypertension", Platelets, 2009
Crossref
8 words — < 1%

22 Lin Shi. "Association of energy-adjusted dietary inflammatory index and frailty in older adults with nonalcoholic fatty liver disease", Experimental Gerontology, 2023
Crossref
8 words — < 1%

23 Mialki, Kaley. "Consumer Acceptability, Family Mealtime Behaviors, and Health Outcomes of a Meal Kit Intervention Designed for Families With Low Income",
8 words — < 1%

University of Florida, 2023
ProQuest

24	Nilgun Seremet Kurklu, Nimet Karatas Torun, Ikbal Ozen Kucukcetin, Aslı Akyol. "Is there a relationship between the dietary inflammatory index and metabolic syndrome among adolescents?", Journal of Pediatric Endocrinology and Metabolism, 2020<br>Crossref	8 words — < 1%
25	academic.oup.com<br>Internet	8 words — < 1%
26	doaj.org<br>Internet	8 words — < 1%
27	journals.plos.org<br>Internet	8 words — < 1%
28	"Abstracts", Diabetologia, 2005<br>Crossref	7 words — < 1%
29	Xuanyang Wang, Jinxia Hu, Lin Liu, Yuntao Zhang, Keke Dang, Licheng Cheng, Jia Zhang, Xiaoqing Xu, Ying Li. "Association of Dietary Inflammatory Index and Dietary Oxidative Balance Score with All-Cause and Disease-Specific Mortality: Findings of 2003–2014 National Health and Nutrition Examination Survey", Nutrients, 2023<br>Crossref	7 words — < 1%

EXCLUDE QUOTES	OFF	EXCLUDE SOURCES	OFF
EXCLUDE BIBLIOGRAPHY	ON	EXCLUDE MATCHES	OFF
