# Supplementary material for: Correlation between dietary inflammation and mortality among hyperlipidemics
Source: Lipids Health Dis. 2023 Nov 28;22:206. doi: 10.1186/s12944-023-01975-0 (PMC10683303; doi:10.1186/s12944-023-01975-0)
Supplement: Supplementary file 7 — Supplementary Material 7 [file 12944_2023_1975_MOESM7_ESM.pdf]

SPRINGER NATURE

Author Services

Editing Certificate

This document certifies that the manuscript

Correlation between Dietary inflammation and mortality among hyperlipidemics

prepared by the authors

Lili Wang, Tao Liu, Qingdui Zhang, Lele Wang, Qiang Zhou, Jing Wang, Hao Miao, Ji Hao, Chunmei Qi

was edited for proper English language, grammar, punctuation, spelling, and overall style by one or more of the highly qualified native English speaking editors at SNAS.

This certificate was issued on **November 14, 2023** and may be verified on the SNAS website using the verification code **A8EC-E309-BCE7-8B8E-4DDE**.

Neither the research content nor the authors' intentions were altered in any way during the editing process. Documents receiving this certification should be English-ready for publication; however, the author has the ability to accept or reject our suggestions and changes. To verify the final SNAS edited version, please visit our verification page at secure.authorservices.springernature.com/certificate/verify.
If you have any questions or concerns about this edited document, please contact SNAS at support@as.springernature.com.

SNAS provides a range of editing, translation, and manuscript services for researchers and publishers around the world.
For more information about our company, services, and partner discounts, please visit authorservices.springernature.com.
